# Adult Neurogenesis and Stroke: A Tale of Two Neurogenic Niches

**DOI:** 10.3389/fnins.2021.700297

**Published:** 2021-08-10

**Authors:** Mihai Ceanga, Mahmoud Dahab, Otto W. Witte, Silke Keiner

**Affiliations:** ^1^Hans-Berger Department of Neurology, Jena University Hospital, Jena, Germany; ^2^Section Translational Neuroimmunology, Department of Neurology, Jena University Hospital, Jena, Germany

**Keywords:** dentate gyrus, subventricular zone, cortex, striatum, doublecortin, cognition, rehabilitation

## Abstract

In the aftermath of an acute stroke, numerous signaling cascades that reshape the brain both in the perilesional zone as well as in more distal regions are activated. Despite continuous improvement in the acute treatment of stroke and the sustained research efforts into the pathophysiology of stroke, we critically lag in our integrated understanding of the delayed and chronic responses to ischemic injury. As such, the beneficial or maladaptive effect of some stroke-induced cellular responses is unclear, restricting the advancement of therapeutic strategies to target long-term complications. A prominent delayed effect of stroke is the robust increase in adult neurogenesis, which raises hopes for a regenerative strategy to counter neurological deficits in stroke survivors. In the adult brain, two regions are known to generate new neurons from endogenous stem cells: the subventricular zone (SVZ) and the dentate subgranular zone (SGZ) of the hippocampus. While both niches respond with an increase in neurogenesis post-stroke, there are significant regional differences in the ensuing stages of survival, migration, and maturation, which may differently influence functional outcome. External interventions such as rehabilitative training add a further layer of complexity by independently modulating the process of adult neurogenesis. In this review we summarize the current knowledge regarding the effects of ischemic stroke on neurogenesis in the SVZ and in the SGZ, and the influence of exogenous stimuli such as motor activity or enriched environment (EE). In addition, we discuss the contribution of SVZ or SGZ post-stroke neurogenesis to sensory, motor and cognitive recovery.

## Clinical Background: Challenges Facing Stroke Survivors

Stroke is an emergency medical condition due to neuronal death ensuing from a reduced blood flow. Either a vascular obstruction (ischemic stroke in over 80% of cases) or a vascular rupture with bleeding within the brain (hemorrhagic stroke) are the offending mechanisms. It results in an acute loss of function in the territory supplied by the affected vessel, manifesting clinically as paresis, speech difficulty, sensory deficits and/or gait disturbance, among other symptoms. The initial cell death by necrosis is followed by a longer period of structural and functional reorganization (Dirnagl et al., 1999; Murphy and Corbett, 2009).

Stroke remains the second-leading cause of death overall (Roth et al., 2020), and the most burdensome neurological disorder in terms of disability-adjusted life years (Feigin et al., 2021). Disability-adjusted life years due to stroke have been constantly increasing since 1990 and were up 32.4% in 2019 (Roth et al., 2020). Studies show that 3 months after a stroke, almost half the patients have persistent functional limitations. A quarter to a third of those affected are dependent on nursing support (Ward et al., 2005; Schneider et al., 2009). Treatment of acute stroke has advanced significantly over the last decade, especially in developed countries, greatly reducing mortality and improving patients’ functional outcomes (Campbell and Khatri, 2020). On the other hand, therapeutic advances targeting post-stroke long-term motor and non-motor sequelae, such as neuropsychiatric disorders, have been worryingly lagging. In contrast to the high prevalence of post-stroke neuropsychiatric disorders and their negative effect on long-term survival, our understanding of their pathophysiology is inadequate, further limiting the development of effective treatments. The overflow of downstream effects erupting after the occurrence of an acute stroke include, among others, energy failure and electrochemical shifts (Dirnagl et al., 1999), metaplasticity (Murphy and Corbett, 2009), the activation of molecular, multi-omics pathways (Montaner et al., 2020), immune responses (Chamorro et al., 2012), as well as neurodegeneration (Castillo et al., 2019).

An intriguing and potentially therapeutically relevant effect of stroke is the robust stimulation of neurogenesis in the adult brain (Gu et al., 2000). Long viewed with skepticism since its first description by Altman and Das (1965), improvements in birth marking techniques vitalized the research into adult neurogenesis, which is now an intensely studied process in both physiological and in pathophysiological conditions such as stroke, epilepsy, traumatic brain injury, sepsis (Bluemel et al., 2021), and others. Two neurogenic niches, the subventricular zone (SVZ) near the lateral ventricles, and the subgranular zone (SGZ) in the dentate gyrus of the hippocampus are widely recognized in multiple species, including mammals and primates (Gage, 2019). The SGZ has also been demonstrated in humans throughout the lifespan (Eriksson et al., 1998; Moreno-Jiménez et al., 2019), although other groups challenged these findings (Sorrells et al., 2018). It has been repeatedly shown that stroke robustly stimulates neurogenesis in the adult brain (Liu et al., 1998). Importantly, markers of neurogenesis have been reported in adult human brain specimens following stroke (Jin et al., 2006) and in Huntington’s disease (Curtis et al., 2003) and even in Alzheimer’s disease, albeit at a lower rate (Jin et al., 2004; Moreno-Jiménez et al., 2019). The use of neurogenesis markers such as proliferation markers (Ki67 or BrdU), immature neuronal markers (DCX, PSA-NCAM) or even mature neuronal markers (NeuN) is crucial to determine the extent of new neuron formation and to make the existing studies comparable. It seems helpful to combine proliferation markers with neuronal markers in order to focus on neurogenesis in addition to describe a form of plasticity itself. While modulating neurogenesis may provide a much-needed therapeutic opportunity of tackling post-stroke neurocognitive deficits, we lack a full understanding of the functional consequences of the increased neurogenesis following stroke, and of the mechanisms that mediate it. Adult-generated granule neurons in the hippocampus have been implicated in cognitive tasks such as hippocampus-dependent memory and pattern separation (Clelland et al., 2009; Garthe et al., 2009; Sahay et al., 2011) as well as in depression and anxiety by mediating the effect of some antidepressants (Encinas et al., 2006), but also by promoting—at least in mice—stress resilience (Snyder et al., 2011). On the other hand, neurons generated in the SVZ have been shown to migrate in the basal striatum and cortex following stroke and have been implicated in sensorimotor recovery. Adult neurogenesis, therefore, stands at the intersection of motor and non-motor long-term complications of stroke. After a short summary of the clinical challenges posed by the long-term neuropsychiatric complications of stroke, we provide a summary of the latest findings on stroke-induced neurogenesis in the two neurogenic niches in the mammal brain.

### Post-stroke Dementia

Acute cognitive impairment in the wake of stroke is well recognized (Del Ser et al., 2005), and cognitive recovery after thrombolysis or thrombectomy is slower than recovery of motor symptoms (Alawieh et al., 2020). It has been estimated that stroke accelerates the development of dementia by about 10 years (Ronchi et al., 2007), but such estimates are limited by confounding factors such as pre-stroke rate of cognitive decline, stroke severity, stroke location, development of depression and coexistence of other pathologies. Nevertheless, several studies addressing the issue of post-stroke dementia reported a persistent and steeper rate of cognitive decline after stroke over a long-term follow-up of 6–12 years, even after correcting for its pre-stroke rate, leading to an independent increase in mortality (Zheng et al., 2019). The Oxford Vascular Study found a high variability in the incidence of post-stroke dementia, depending on clinical characteristics of stroke, but also on pre-stroke risk factors. Still, 1 year after stroke the prevalence of dementia was accelerated by 25 years in those suffering a severe stroke, by 4 years in those suffering a minor stroke and by 2 years in patients experiencing a transient ischemic attack (Pendlebury and Rothwell, 2019). Despite its toll on quality of life and even on mortality risk, the pathophysiology of post-stroke dementia is not understood (Mijajloviæ et al., 2017).

### Post-stroke Depression and Other Neuropsychiatric Symptoms

Other underrecognized and undertreated complications of stroke are represented by neuropsychiatric disorders encompassing depression, anxiety, apathy, personality change, and post-traumatic stress disorder (PTSD). These occur in about one third of stroke survivors and significantly reduce the quality of life as well as increase the rate of institutionalization [for a review see Ferro et al. (2016)]. Post-stroke depression occurs in up to 40% of cases (Robinson et al., 1987) and negatively influences the recovery of activities of daily living in stroke survivors (Parikh et al., 1990). Stroke location seems to play a role in post-stroke depression, with left anterior strokes possibly posing a risk factor for both major and minor depression (Robinson et al., 1984). The mortality risk of patients with post-stroke depression was found to be 3.5-fold increased during a follow-up of 10 years (Morris et al., 1993). Antidepressant treatment given within the first 6 months post-stroke had a positive effect on long-term survival in both depressed and non-depressed patients (Jorge et al., 2003). Despite the availability of better treatment options for post-stroke depression, it remains significantly underrecognized and undertreated (Medeiros et al., 2020). In conclusion, long-term non-motor complications of stroke influence significantly and negatively both the quality of life and the survival of stroke patients. However, despite their clinical relevance, the underlying pathophysiological mechanisms are largely unknown, limiting therapeutic opportunities.

## Physiology of Two Neurogenic Niches – General Principles of Organization

### The Subventricular Neurogenic Niche

**FIGURE 1:**
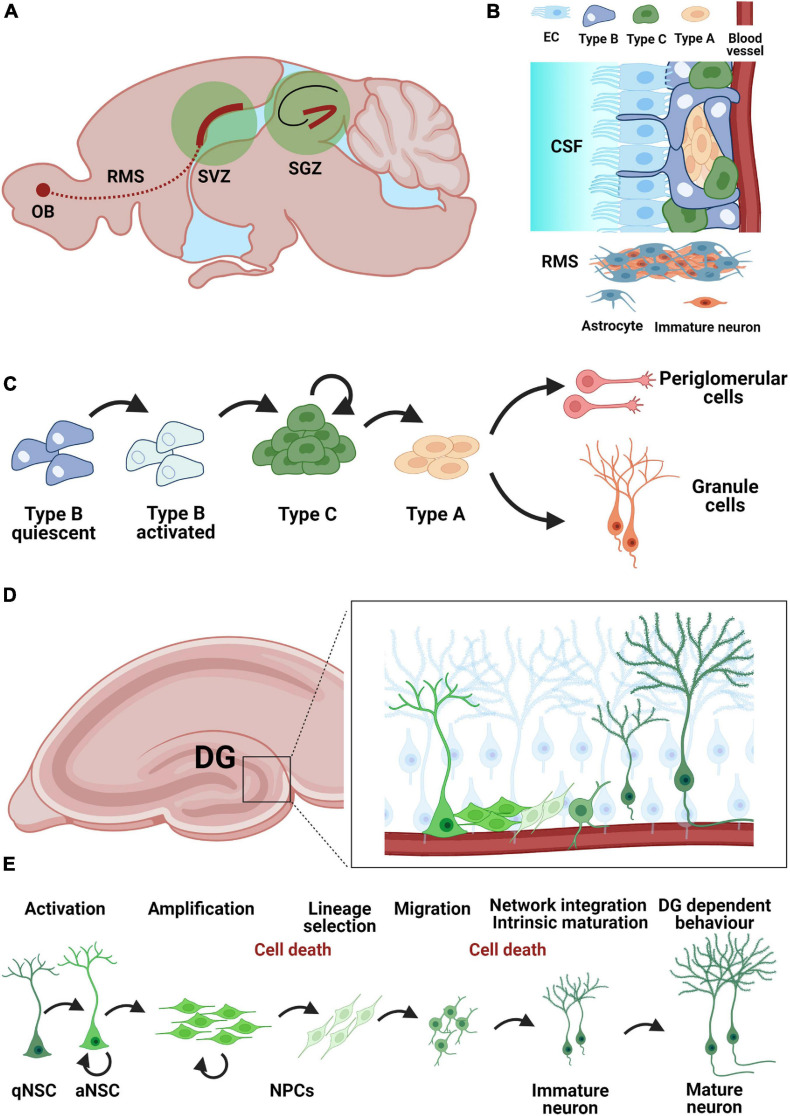
Neurogenic niches in the adult mouse brain. **(A)** Neurogenic niches in the adult brain include the subventricular zone (SVZ) and the subgranular zone (SGZ) of the hippocampal dentate gyrus. Immature neurons from the SVZ migrate along the rostral migratory stream (RMS) toward the olfactory bulb (OB) **(B)** Organization of the SVZ neurogenic niche. Neural stem cells (Type B) contract both the cerebrospinal fluid (CSF) *via* ependymal cells (EC) and blood vessels as a mechanism for humoral regulation. Immature neurons migrate along the RMS using astrocytes as guides. **(C)** Developmental steps during neurogenesis in the SVZ: quiescent astrocytic stem cells (Type B) in the SVZ become activated and give rise to neuroblasts (Type A) *via* a pool of transient-amplifying cells (Type C). Neuroblasts can give rise to granule cells and periglomerular cells. **(D)** Adult neurogenesis in the dentate gyrus**:** localization of the SGZ neurogenic niche within the hippocampus. **(E)** Adult neurogenesis in the dentate gyrus arises from a population of quiescent neural stem cells (qNSC) with astrocytic characteristics Activated neural stem cells (aNSC) give rise to a pool of amplifying neural precursor cells (NPCs), which further undergo lineage selection, migration, morphological and functional maturation as well as network integration before becoming mature granule cells by about 8 weeks post-mitosis. The survival of adult born granule cells is regulated during an early (24–48 h post-mitosis) and a late phase (12–16 days post-mitosis) mostly through apoptosis.

One of the two established regions where neural stem cells (NSCs) are activated throughout life and contribute to the formation of new neurons is the SVZ of the lateral ventricles (Figures 1A–C). New neurons undergo different stages from stem cells to immature neurons, such as proliferation, lineage selection, migration, survival, integration, and functional maturation. These stages partially overlap and are interdependent. The resident NSCs also called type B cells are predominantly in a resting state, have a radial-glial like morphology and show formation of vascular endings with the blood vessels in the SVZ. In addition, NSCs also possess an apical end that extends through the ependymal layer and makes contact with the cerebrospinal fluid in the lateral ventricles (Figure 1B). Type B cells have the ability to generate neurons as well as astrocytes and oligodendrocytes (Ahn and Joyner, 2005; Menn et al., 2006). Clonal analysis and fate mapping of single NSCs found that these cells present a limited capacity for self-renewal, being exhausted in a few weeks. Their progeny, however, show a particularly fast lineage amplification (Calzolari et al., 2015). The process of neurogenesis starts with type B cells (Merkle et al., 2004; Bonaguidi et al., 2016) which form transient amplifying type C cells by asymmetric cell division. Type C cells in turn form neuronal progenitor cells (neuroblasts, type A cells). Before these neuroblasts differentiate into olfactory granule cells (GCs) and periglomerular cells, they must migrate a long distance along the rostral migratory stream toward the olfactory bulb (OB) (Luskin, 1993). During migration, they form a chain consisting of a group of 30–40 cells which lie close together and are encased by astrocytes (Figures 1B,C). After about 5 days, the neuroblasts reach the OB, detach from the chain in the RMS and begin to migrate to their final positions to reach either the granule cell layer (GCL) or the glomerular layer (GL) (Luskin, 1993; Lois and Alvarez-Buylla, 1994; Lois et al., 1996). The migration of neuroblasts along the RMS can be classified into tangential migration, which runs from caudal to rostral and requires close interaction of the neuroblasts with the astrocytes that envelop them; and radial migration in the OB, which runs independently of the astrocytes. Another crucial point in adult neurogenesis is the regulation of newborn cell survival. To ensure that a stable and adequate number of new OB neurons is available, some newly formed cells must be eliminated. This occurs *via* programmed cell death and is also critical for maintaining orderly migration along the RMS (Kim et al., 2007). Surviving neuroblasts differentiate into one of the subtypes of OB interneurons. The OB is constructed in an orderly fashion. For example, the hair cells are located in the olfactory epithelium and send their axons to the first switching region of the OB, the glomeruli (GL). Each hair cell has a specific scent receptor represented by a glomerulus. The glomeruli are surrounded by periglomerular interneurons, most of which use the neurotransmitter gamma-amino-butyric acid (GABA) or dopamine (Lledo et al., 2005; Wachowiak and Shipley, 2006). The information from the hair cells is transmitted in the glomeruli to mitral and tufted cells, which lie with their cell somata in the mitral/tufted cell layer. Both cell types are headmaster neurons of the OB, which are interconnected by GCs whose cell somata lie in the GCL. The final step in the formation of new neurons in the adult brain is synaptic integration into the existing neuronal network. The regulation of the integration and thus the survival of the newly formed neurons in the OB is primarily regulated by sensory signals *via* the hair cells. The principle in effect here is “use them or lose them.” This means that new neurons that are not integrated into the neuronal network of the OB die (Corotto et al., 1994). By enriching the environment with odorants [odor enriched environment (EE)], the survival of the newborn cells can be positively influenced (Rochefort et al., 2002; Lledo and Saghatelyan, 2005; Grelat et al., 2018; Li et al., 2018), albeit this is only possible during a critical period (Yamaguchi and Mori, 2005). During this period, the neurons already receive synaptic inputs from neighboring cells (Petreanu and Alvarez-Buylla, 2002). All these regulatory mechanisms must cooperate seamlessly to form new functional neurons in appropriate numbers for the OB. In addition to the SVZ, studies report low-level constitutive neurogenesis in specific regions of the adult neocortex under physiological conditions (Gould and Gross, 2002). The studies in human post-mortem tissue showed proliferation and migration of neuroblasts taking place at least up to the age of 13 (Paredes et al., 2016; Sorrells et al., 2018). However, direct evidence of neurogenesis in the OB is still not clear. Nevertheless, human studies suggest that neurons can be formed in the striatum, a structure that plays an important role in cognition and motor function and is close to the SVZ (Ernst et al., 2014).

### The Hippocampal Neurogenic Niche

The second major neurogenic niche in the adult brain is the SGZ of the hippocampal dentate gyrus, where adult born granule cells (abGCs) are produced, mature and integrate into the existing network (Figures 1A,D,E; van Praag et al., 2002), for a review see Denoth-Lippuner and Jessberger (2021), in a process that recapitulates the maturation milestones of fetal neurogenesis. Local NSCs are identified by their astrocytic molecular characteristics (e.g., expression of glial fibrillary acidic protein, GFAP) and radial glia-like (RGL) morphology, resembling embryonal neural progenitors, and have the capacity for self-renewal and multipotency (Bonaguidi et al., 2011; Bottes et al., 2021). abGCs undergo five developmental stages during adult hippocampal neurogenesis: (1) activation of quiescent RGLs in the SGZ; (2) amplification of non-radial precursor and intermediate progenitors; (3) generation of neuroblasts through lineage selection; (4) migration of immature neurons and (5) integration and maturation of adult-born dentate GCs (Figure 1E). NSC (type 1 or RGL cells) are predominantly found in a quiescent state (qNSC) and upon activation (aNSC) can divide asymmetrically to generate an amplifying population of non-RGL progenitor cells (NR cells or type 2 cells). Type 2 cells can be differentiated based on the expression of different proteins into type 2a and 2b, with type 2b cells beginning to show a neuronal lineage. These cells divide mostly symmetrically, but also asymmetrically (Encinas et al., 2011; Pilz et al., 2018) to generate type 3 cells, which are already committed to the neuronal lineage. Most cells will be removed in a two-step process within the critical first 3 weeks after birth (Cameron et al., 1993; Kempermann et al., 2003; McDonald and Wojtowicz, 2005; Mandyam et al., 2007; Sierra et al., 2010). The larger part of cell removal happens early, 24–48 h post-mitosis (Pilz et al., 2018) through apoptosis. The second stage of cell death occurs during the phase of network integration, when glutamatergic NMDA-type input is essential for cells survival (Tashiro et al., 2006). Once the neurons have survived, they are integrated into the existing network indefinitely (Dayer et al., 2003; Kempermann et al., 2003). The newly formed abGCs are excitatory neurons with a characteristic set of functional properties including enhanced plasticity (Schmidt-Hieber et al., 2004). Synapse formation is the basis of network integration as new neurons appear to compete with old GCs for the available axonal boutons (Ge et al., 2008). The establishment of first synapses occurs once the cell body of the developing neuron is in the granular zone and its dendrites grow into the molecular zone (Toni and Schinder, 2015). Synaptogenesis takes several weeks, during which the number of spines increases with dendrite length. The dendrites of the new neurons branch toward the molecular layer as the axon moves into the CA3 region (Hastings and Gould, 1999). Within 8 weeks, a newly formed GC is integrated into the pre-existing neural network, although this process is prolonged in higher mammals and may take over 6 months to complete in humans (Kohler et al., 2011). Finally, the integration of the new neurons leads to functional modification of the preexisting network (Ikrar et al., 2013). Adult neurogenesis is important for cognition and its plasticity (Kempermann et al., 2015). Particularly involved are learning, spatial memory and the ability to distinguish similar experiences (Clelland et al., 2009; Sahay et al., 2011), which is referred to as pattern separation. The need to adapt to new environments is thought to underlie the continuous generation of new neurons in the adult hippocampus, a hypothesis supported by the finding that adult hippocampal neurogenesis is enhanced by environmental factors such as an EE or running (Kempermann et al., 1997). However, more specific tasks such as grasp training also leads to a bilateral increase in neurogenesis in the rat hippocampus (Wurm et al., 2007). Adult neurogenesis in the hippocampus is also affected by disease states and may even actively change the disease course (Toda et al., 2019) by inducing aberrant neurogenesis (Parent et al., 1997).

## Stroke and Adult Neurogenesis

**FIGURE 2:**
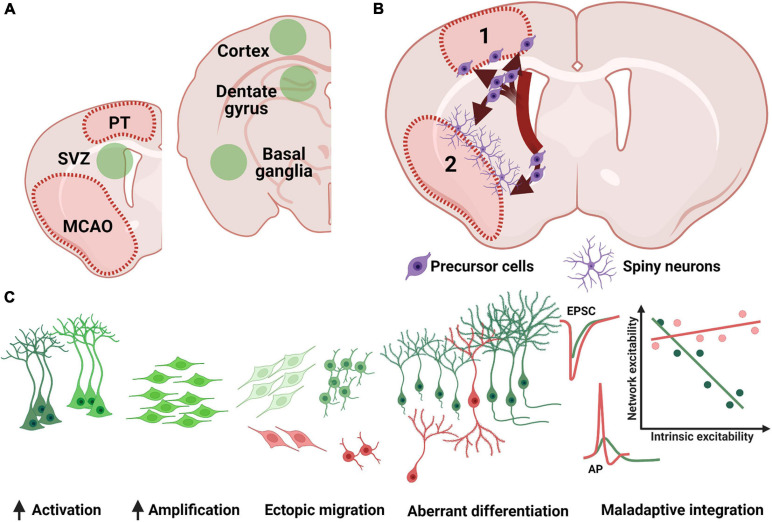
Remote effects of stroke on adult neurogenesis. **(A)** Post stroke neurogenesis (green fields) has been detected in both canonical neurogenic niches (SVZ, subventricular zone; SGZ, subgranular zone) as well as in previously non-neurogenic regions (e.g., cortex and basal ganglia). **(B)** Increased SVZ proliferation is followed by ectopic migration in the cortex (1) or basal ganglia (2), where precursor cells are detected at the periphery of the lesion and develop into spiny neurons in the basal ganglia or form a more heterogenous cell group the cortex (see text for details). **(C)** SGZ neurogenesis shows a robust increase in cellular proliferation, although a subgroup of newly generated neurons (red cells) has been shown to migrate ectopically into the hilus, or otherwise present morphological (bipolar cells or changes in dendritic arborization) or functional (accelerated and uncoupled intrinsic and synaptic maturation) abnormalities. PT, photothrombosis; MCAO middle cerebral artery occlusion; EPSC, excitatory post-synaptic currents. AP, action potential.

Stroke triggers changes not only in the brain territory afferent to the affected blood vessel, but also in brain areas that are distant from the infarct core, as may be the case with the hippocampus (Keiner et al., 2010). The previously described developmental steps of proliferation, migration and differentiation are differentially affected in the two main neurogenic niches post-stroke (Figure 2). Due to niche-specific nature of adult neurogenesis’s response to ischemia, as well as the different functionality of the two neuroanatomical regions where neurogenesis occurs, the overall behavioral effect of stroke-induced neurogenesis can differ not only by functional modality (e.g., motor performance vs. cognitive performance) but also by the very direction of the adaptative effect (maladaptive vs. beneficial). Consequently, stroke-induced neurogenesis is a at privileged position to influence both motor and neuropsychiatric performance and explain the development of motor, cognitive and neuropsychiatric sequelae of stroke by telling “a two-faced tale.”

In stroke research, rodent models that cause permanent or transient occlusion of the middle cerebral artery are often used. Using the transient occlusion of the middle cerebral artery, infarct volume and location is a function of time, which can be adjusted to suit the scientific question at hand. This flexibility in stroke severity is counterbalanced by its disadvantage, consisting in a long practice to achieve sufficient reproducibility (Ceanga et al., 2021). When inducing larger infarcts, the mortality increases starting 24 h after stroke induction. Another important method of inducing a cortical stroke is by photothrombotic infarcts. These infarcts have the advantage that the size and location of the cortical lesion can be freely selected, and striatal or other subcortical lesions can be prevented. Furthermore, the method is less invasive and more reproducible. Using the photothrombotic model, cortical neurogenesis could thus be specifically investigated in more detail. The decisive factor here is that very specific areas of the cortex can be damaged by a photothrombotic stroke. Due to the circumscribed damaged area, it is possible to analyze in detail the changes in the peri-infarct environment, such as the migration of newly formed progenitor cells or the presence of new mature neurons, but also after therapeutic interventions such as anti-inflammatory treatments or rehabilitative training (Keiner et al., 2008, 2009; Liebigt et al., 2012).

### Post-stroke Striatal und Cortical Adult Neurogenesis

The induction of cerebral ischemia is accompanied by stimulation of endogenous neurogenesis (Kee et al., 2001). Several studies have described the heterogeneous nature of SVZ stem cells and their potential to contribute to the generation of new striatal neurons (Suzuki and Goldman, 2003; Menn et al., 2006). In this context, previous studies could show that SVZ cells generated after a lesion show an altered migration path, including toward the striatum and cortex (Inta et al., 2008). Ischemia thus leads to an induction of cell renewal and migration of young neurons to areas where initially no detectable neurogenesis takes place under physiological conditions (Figures 2A,B; Jiang et al., 2001). Interestingly, stroke-induced animal models generally show increased neurogenic potential in the striatum. This has been suggested as a method for self-repair following an ischemic event affecting this area. Doublecortin positive (DCX^+^) cells are the main heralds of neurogenesis in the cortex and striatum, and they often serve as a surrogate for the immunohistological detection of adult neurogenesis. Due to the formation of DCX^+^ cells in the SVZ and their clear ability to migrate, these neuronal cells are probably the most important source of new neurons in the cortex and striatum. Finally, DCX^+^ cells are the precursors of mature neurons, which are often difficult to detect and have a low survival rate. The focus of the following sections is therefore mainly on the DCX^+^ cells.

Following stroke, endogenous stem cells are activated in the SVZ and, after asymmetric cell division, DCX^+^ neuroblasts are generated and migrate into the striatal or cortical stroke area away from the rostral migratory stream. These DCX^+^ neuroblasts are of great interest, as they already show neuronal determination and thus demonstrate an increased potential to differentiate into neurons (Brown et al., 2003). DCX has been identified as a microtubule-associated protein expressed by neuroblasts during cell migration to the brain surface and during a limited growth phase, in both developing and adult mammals (Francis et al., 1999). DCX thus plays a crucial role in neuronal migration during embryonic and postnatal development by being involved in phosphorylation-dependent microtubule stabilization (Gleeson et al., 1999), nuclear translocation (Koizumi et al., 2006) and growth cone dynamics (Koizumi et al., 2006). A special role was assigned here to the protein doublecortin, which according to immunohistochemical studies is characteristically expressed by immature neurons in the neurogenic regions (hippocampus and SVZ). For these reasons, DCX is also widely used as a marker for neuronal progenitor cells and for neurogenesis. The combination of different proliferation and glial markers seems to be helpful to better describe neurogenesis *per se*. In the mature adult brain, expression of DCX outside the neurogenic areas in the hippocampal formation and SVZ is rather rare (Brown et al., 2003). Functionally however, DCX seems to be dispensable for the generation of abGCs (Merz and Lie, 2013). Under pathophysiological conditions, such as stroke, the expression pattern of DCX changes and DCX^+^ cells can be detected in cortical and striatal areas (Liu et al., 2009) outside the circumscribed neurogenic regions (Arvidsson et al., 2002). In this context, Arvidsson et al. (2002) demonstrated a significant increase in proliferation and striatal recruitment of SVZ neuroblasts after focal ischemia in rats using double labeling with Bromodeoxyuridine (BrdU) and DCX, or NeuN (a common immunohistochemical marker of mature GCs). BrdU and DCX labeling confirmed the neuronal fate of these cells, which developed into GABAergic interneurons (Dayer et al., 2005). Further studies followed and were able to demonstrate that the post-ischemic newly formed neurons integrate into the network. This was demonstrated by the expression of immediate early genes (c-fos), and the detection of action potentials. Accordingly, it is postulated that the DCX^+^ neuroblasts diverge from the RMS and migrate into the striatum or cortex and do not reach the OB, but instead complete their differentiation and maturation in the striatum (Bédard et al., 2002). After stroke, a transient increase in DCX^+^ neuroblasts, as well as long chains of migrating neuroblast associated with striatal blood vessels were detected. These migrating neuroblasts matured, expressed presynaptic vesicles and formed synapses within the striatal microcircuit (Yamashita et al., 2006). However, it was shown that new migrating SVZ neurons were associated with both newly formed as well as old blood vessels in the striatum after stroke (Kojima et al., 2010). The newly generated GABAergic and cholinergic striatal neurons develop dendrites and spines, and show electrophysiological properties indicative of full integration into the pre-existing neuronal network of the striatum (Hou et al., 2008). Furthermore, Magnusson et al. (2014) found that stroke induces a Notch1 gated neurogenic differentiation in striatal astrocytes.

In summary, previous studies show that endogenous progenitor cells are capable of partial neuronal regeneration in the striatum. However, the number of these newly formed neurons is extremely low, at least in epilepsy, and a therapy focused on neuronal restitution does not appear to be sufficient so far (Parent et al., 1997). Furthermore, the functional relevance of endogenous neurogenesis is unclear due to the reduced survival of newborn cells in the striatum (Arvidsson et al., 2002). All the open questions aside, one main takeaway is that ischemia-induced neurons as well as neuroblasts can migrate into the striatum and differentiate into new neurons post-stroke (Arvidsson et al., 2002).

In addition to the striatum, several studies have found evidence that the cortex may also be capable of regeneration under certain conditions (Magavi et al., 2000), including after ischemia, albeit with a low number of newly formed neurons (Kaplan, 1981). In contrast, others have failed to demonstrate cortical neurogenesis after focal ischemia (Kornack and Rakic, 2001). For some of these studies, photothrombotic models of infarct induction were used in addition to the middle cerebral artery occlusion model (MCAO). Studies have shown that after photothrombotic infarcts, cells in the perilesional cortex—beyond the circumscribed neurogenic regions—express DCX. These DCX^+^ cells could be detected mainly in the area of the sensorimotor cortex of the small infarcts. The number of DCX^+^ cell in the perilesional zone can be increased by external pharmacological manipulations such as application of growth factors (Pencea et al., 2001). A closer look at these DCX^+^ cells revealed that these cortical cells presented as a heterogeneous group as two distinct subpopulations can be found around the infarct area (Kunze et al., 2015) characterized DCX^+^ cells with a star-shaped and bipolar morphology. The star-shaped cells were ubiquitously and homogeneously distributed throughout the cortex around the peri-infarct area and expressed typical glial markers such as GFAP and S100beta in addition to the neuronal marker DCX. Several studies have shown that glial cells have the ability to express DCX (Kunze et al., 2015; Keiner et al., 2017). In addition, an *in vivo* study demonstrated that by antagonizing Olig2 or overexpressing Pax6 through intraparenchymal injections of retroviral vectors, a significant proportion of astrocytes adopted a neuronal phenotype and were characterized by DCX expression. In addition, it was possible to detect post-synaptic currents in some of the transduced cells after Pax6 overexpression (Kronenberg et al., 2010). The extent to which these star shaped DCX^+^ glial cells can form mature neurons post-stroke is still unclear. It has also been shown that, under physiological conditions, NG2 + cells (polydendrocytes/OPCs) express DCX in the cortex (Keiner et al., 2017). In addition to generating oligodendrocytes, NG2 glia may have the potential to generate a variety of cell types such as astrocytes and neurons, both *in vitro* and *in vivo* at postnatal or adult stages (Belachew et al., 2003), although the latter has not yet been fully elucidated. Heterogeneity within the NG2 cell population may play an important role here, as localization and developmental factors regulate their activity and progeny. Direct evidence of the extent to which oligodendrocyte progenitors expressing NG2 and DCX have the capacity for cortical post-ischemic neurogenesis has, to our knowledge, not yet been demonstrated.

In addition to the star shaped phenotype of DCX^+^ cells, (Kunze et al., 2015) were able to demonstrate a subtype with bipolar morphology in the area surrounding the infarct. These DCX^+^ cells showed a bipolar morphology corresponding to the DCX neuroblasts from the RMS. It can be assumed that these neuroblasts migrate from the SVZ into the infarct zone. Interestingly, this bipolar phenotype is exclusively found at the inferior border of cortical infarcts, adjacent to the corpus callosum, and is not distributed across the cortical layers of the infarct. It is suspected that both stellate and bipolar DCX^+^ cells may contribute to cortical neurogenesis. While many of the DCX^+^ neuroblasts in the peri-infarct environment persist for several months, no mature NeuN + neurons were shown to result from induction of cortical infarction alone. However, other protocols of cortical lesions may induce local neurogenesis with neuronal maturation and long-lasting stable network integration (Magavi et al., 2000) induced synchronous apoptotic degeneration of layer VI corticothalamic projection neurons by targeting neuronal degeneration with chromophores. This study suggests that neural progenitor cells *in situ* have the potential to differentiate into mature neurons. Despite these extensive studies, several questions remain open, such as which DCX^+^ cells contribute to post-lesional neurogenesis and which stroke-induced factors trigger the altered migration of neuroblasts into the infarct area. The existence of multipotent neural progenitor cells in the neocortex, having the potential to differentiate into mature neurons, has already been demonstrated in several studies (Kondo and Raff, 2000). Following ischemic lesions of the frontoparietal cortex in adult rats, an increase in neuroblasts in the SVZ, rostral migratory stream, corpus callosum and perilesional zone after stroke induction was observed (Gotts and Chesselet, 2005). Ohab et al. (2006) demonstrated migration of DCX^+^ cells from the SVZ in to the peri-infarct zone of cortical infarcts in the sensorimotor barrel field cortex. In addition to migration of neuroblasts from the SVZ toward the lesion, it is also conceivable that quiescent progenitor cells in the neocortex are stimulated during an infarct and contribute to the population of DCX^+^ cells in the perilesional zone.

Remarkably, the number of neuroblasts in the infarct environment positively correlates with the recovery of functional deficits after stroke. Ablation of DCX^+^ neuroblasts worsened functional recovery (Jin et al., 2010). These results suggest that ischemia-induced neurogenesis has a significant contribution to functional recovery after stroke. In addition, Keiner et al. (2009) demonstrated that BDNF application increases the number of neuroblasts but does not improve functional sensorimotor recovery. Comparable to these findings, (Schäbitz et al., 2007) found improved motor performance in the Rotarod test, bar balance test and neurological score after post-stroke intravenous BDNF application. However, given the low number of new neurons in the perilesional zone, it seems unlikely that DCX neuroblasts alone contribute significantly to the improvement in functional performance.

In summary, animal studies show that deviant migration from the SVZ into the cortical regions after crossing the corpus callosum is possible. Besides migration, the local generation of new cortical neurons after ischemia only seems possible under special conditions, such as application of various exogenous factors that promote both the differentiation of DCX neuroblasts as well as their survival. Furthermore, it seems very likely that the DCX^+^ neuroblasts are able to form neurons in the area of the striatum.

### Post-stroke Hippocampal Adult Neurogenesis

Similar to the SVZ, pathological processes such as cerebral infarctions also lead to a robust increase in adult neurogenesis in the hippocampal dentate gyrus (Figure 2C; Liu et al., 1998). Both, large cortico-subcortical infarcts, as well as small cortical infarcts, lead to an increase in the rate of neurogenesis, even if not localized in the hippocampal region (Kluska et al., 2005). However, the increase in neurogenesis tends to be higher for larger infarcts, as induced by temporary occlusion of the middle cerebral artery, compared to small, narrowly circumscribed infarcts, such as those induced by photothrombosis (Niv et al., 2012). Not only the ipsilateral hemisphere is affected, but also the contralateral one (Jin et al., 2001; Cuartero et al., 2019). Numerous studies have shown that the proliferation, migration, and differentiation of neuronal progenitors, as well as their survival (Arvidsson et al., 2001) and integration (Niv et al., 2012) are influenced by the induction of a stroke, which in turn affects neurocognitive functions (Yagita et al., 2001). The exact dynamics of the increase in neurogenesis may be dependent on the ischemia model used. However, this increase in cell number after ischemia can be evident as early as 5 days after infarction (Tanaka et al., 2004). Even within the first 6 h, the number of type-1 and type-2a cells increases significantly after photothrombotic infarction (Kunze et al., 2006). However, at postischemic examinations after 24 and 72 h, a significant increase in type-2b and type-3 cell types can be seen, whereas the number of type-1 and type-2a cells is now decreased. In addition to changes in progenitor populations, an increased number of newly generated neurons 6 weeks after induction of small cortical (Geibig et al., 2012) and large cortico-subcortical infarcts was demonstrated (Kluska et al., 2005). In general, already a few days after the infarction the division rate of the progenitor cells is greatly increased, while the phagocytosis of newly generated precursor cells is not impaired (Rudolph et al., 2021). This high division rate already starts to decrease in the second week and falls back to the control level within several weeks (Liu et al., 1998; Arvidsson et al., 2001; Yagita et al., 2001; Cuartero et al., 2019). However, this sharp increase in the progenitor population only caused a 50% increase in neurogenesis at 4 weeks post-ischemic compared to control animals (Kluska et al., 2005; Walter et al., 2010; Sun et al., 2019). This means that the progenitor population is lost during differentiation and maturation into the integrated neuron. In this context, it is not yet clear at which checkpoint and by which mechanism the new progenitor cells decrease. The maturation and integration process of the neuronal precursor cells seem to play an important role. Using both cortical photothrombosis and the MCAO model of stroke, (Niv et al., 2012) studied the migration and morphological maturation of retrovirally labeled newborn GCs. Newborn neurons were labeled 4 days after stroke and morphological analysis was performed 6 weeks later. Most neurons (ca. > 90%) displayed normal migration along the width of the GCL and gross dendritic development, albeit with an increased total dendritic length and a higher number of apical dendrites. However, there was a high rate (5–10%) of migration or morphological abnormalities after stroke, as evidenced by ectopic neurons within the CA4 or by bipolar cells, showing an additional basal dendrite. These morphological abnormalities appeared more often in the MCAO group, which also caused a significantly larger infarct volume, without a concurrent change in hippocampal volume. Importantly, both morphologically normal neurons as well as neurons with aberrant morphology (aberrant neurogenesis) displayed dendritic spines, which suggests network integration and functional recruitment. Spine density was increased after MCAO in morphologically normal, but not aberrant neurons, and the presence of mushroom spines (ca. 30 and 45% of spines in morphologically normal and aberrant neurons, respectively) indicated stable network connections (Niv et al., 2012). Quite different results were reported by Sheu et al. (2019). Retrovirally labeled adult-generated GCs in the mouse hippocampus 1 week after an MCAO or sham operation were investigated for morphological development every week for up to 4 weeks after stroke. While there were no changes in the migratory pattern after stroke, a decrease in total dendritic length and number of branches was seen starting from the second week after stroke. Dendritic spines were also reduced starting from the third week after stroke. These changes were long-lasting, as the authors found them in neurons born up to 21 days after stroke. More detailed evidence of aberrant neurogenesis was recently provided by Cuartero et al. (2019) by investigating the morphology of post-stroke generated neurons both ipsilateral but also contralateral to the ischemic lesion. A differential effect was noted in the length of apical dendrites, with ipsilateral neurons more often displaying reduced branching and dendritic length, while contralateral neurons showed enhanced branching and increased dendritic length.

In addition to the presence of spines, the study by Geibig et al. (2012) demonstrated expression of the immediate early gene c-fos in the newly formed neurons 8 weeks after stroke as an indication of functional recruitment. Their activation was context-specific, greater with “spatiotemporal” tasks than with “sensorimotor” tasks (Geibig et al., 2012). While ladder run or EE increase the total number of newly generated neurons, they do not increase the rate of functional recruitment (Geibig et al., 2012). The functional maturation of stroke-induced GCs was investigated by Ceanga et al. (2019). Adult born GCs mostly recapitulate the stages that developing neurons undergo during embryogenesis. During cell maturation, abGC coordinate their high intrinsic excitability (depolarized resting potential, high membrane impedance, low rheobase and high gain) to the low excitatory input due to incomplete network integration. Increasing excitatory synaptic drive is offset by a decrease in intrinsic excitability (Schmidt-Salzmann et al., 2014). Two weeks after stroke, immature, DCX^+^ GCs showed an accelerated maturation of passive properties such as a lower input resistance, resting potential and membrane time constant, and were more likely to fire fast, brief action potentials characteristic of mature neurons (Ceanga et al., 2019). Network excitability was also increased, with post-stroke abGC showing larger AMPA currents but no increase in the frequency of excitatory currents. Paired pulse facilitation after stimulation of the lateral perforant path was also unchanged, suggesting post-synaptic, but not presynaptic changes. The coupled maturation of intrinsic and network excitability usually present during normal GC development was significantly impaired, resulting in intrinsically hyperexcitable neurons receiving strong network excitation (Figure 2C, last panel). Of note, none of the investigated neurons showed an overtly aberrant morphology. In addition to the typical post-stroke changes in the developmental stages of proliferation, migration, differentiation, and integration, the long-term changes and the direct influence of old age are of decisive importance, especially with regard to everyday clinical life. Several studies, both in rodents (Driscoll et al., 2006) and in humans (Knoth et al., 2010) have shown that adult neurogenesis decreases with age (Moreno-Jiménez et al., 2019). Similarly, the timing of a stroke plays a major role in the formation of new neurons. Using animal models, it has been shown that in 16-month-old rats after focal ischemia, adult neurogenesis is significantly reduced in the SGZ of the hippocampus compared with young rats at 3 months of age (Darsalia et al., 2005; Walter et al., 2010). In addition to the age-related decrease in all progenitor cells after a stroke, the proliferation rate also shifts. While in young mice type 1 and type 2b cells are stimulated to proliferate, aged mice only show an increase in type 2a progenitor cells (Walter et al., 2010). Finally, stroke in old age does not lead to an increase in neurogenesis as it does in young animals. In addition to the direct comparison of stroke-related changes between old and young, Kathner-Schaffert et al. (2019) investigated the long-term effects of stroke over a time period of about 15 months. The study demonstrated a significant decrease in endogenous proliferation of Ki67-positive cells 6 months after stroke. Moreover, the study showed a strong reduction in adult neurogenesis 4.5 and 6 months after stroke induction in 3-month-old animals. Perfusions were performed at 6, 7.5, and 9 months of age, with the proliferation marker BrdU applied 6 weeks earlier. Interestingly, the total number of new neurons fell below the control level. In addition to the cellular changes, 7.5- and 9-month-old animals showed reduced use of hippocampus-dependent strategies, especially in the re-learning phase.

In summary, stroke induces a sharp but transient increase in SGZ neurogenesis, which is proportional to the size of the infarct. However, only about a half of newly generated cells survive in the long term to integrate in the preexisting neural network, and a subgroup of these neurons will show abnormal morpho-functional development, including aberrant migration, bipolar morphology, or an abnormal functional development. Finally, there is also evidence of a long-term malfunction of the neurogenic niche following the immediate post-stroke neurogenesis burst.

## Rehabilitative Training and Post-Stroke Neurogenesis

**FIGURE 3:**
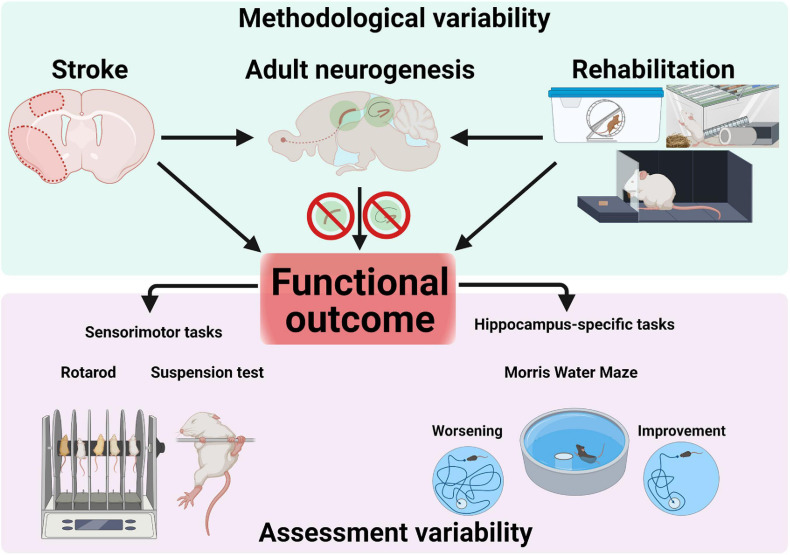
Sources of variability in research on adult neurogenesis after stroke. Methodological factors (e.g., stroke protocol, size and location), rehabilitative interventions (e.g., running, enriched environment or forced reaching training) as well as pharmacological/genetical manipulations blocking adult neurogenesis converge and interact to influence functional outcome. Assessment of functional outcomes assumes using appropriate, specific tests to probe sensorimotor or cognitive performance. Confounding results can occur through the use of unspecific tests (e.g., not probing hippocampus-dependent memory).

The regeneration of motor functions after cerebral ischemia during neurological rehabilitation is based on the one hand on the plastic properties of the central nervous system and on the other hand on compensatory strategies to regain lost functions (Girard et al., 2014). In addition to medication, rehabilitative training of the affected body parts is one of the most important therapeutic measures after a stroke (Biernaskie and Corbett, 2001). The influence of rehabilitative training on cellular plasticity in the area surrounding the infarct and the extent to which these changes can be correlated with improved functional performance will be discussed in the following (Figure 3).

### Rehabilitative Training and Forebrain Neurogenesis

After a stroke, a window of increased neuroplasticity is present for the first weeks. This is characterized, for example, by the changes in dendrites, synapses, neurogenesis, and astrogliosis, as well as the upregulation of growth-promoting genes and proteins (Carmichael et al., 2005). This period seems ideal for interventions such as rehabilitative training, as changes induced by rehabilitative training are simultaneously incorporated into these processes in addition to stroke-induced changes. Consequently, the early transfer of the animals to an EE promotes motor activity and stimulates cognition during this critical period.

Transfer to a stimulus-rich environment, containing for example, running wheels, tunnel systems or climbing possibilities represents a well-established model for modulating cellular and structural plasticity (Biernaskie and Corbett, 2001). In this context, previous studies have shown that exposure to novel environmental factors increases the complexity of neuronal cell dendritic trees and likewise synaptic density or spine length (Greenough et al., 1973). Furthermore, changes in vascularization and cortical layer thicknesses have been described (Black et al., 1990). An EE additionally alters the number of glial cells (Diamond et al., 1966) and specifically promotes survival of newly born neurons in the neurogenic niches (Kempermann et al., 1997; Kronenberg et al., 2003; Fabel et al., 2009). Typically, the EE is equipped with numerous stimulating objects. For rodents, EE is simply changing the standard cage environment which rodents used into more complex housing cages. This can be done by adding toys, running wheels, tunnels and more nesting materials (Fares et al., 2013). Furthermore, the EE is characterized by an increased group size to stimulate social interactions. Thus, an EE promotes non-specific physical activity, social interactions, and exploratory behavior. After stroke, the transfer to an EE results in migration of neural DCX^+^ progenitor cells in the striatum in the peri-infarct region (Komitova et al., 2005; Venna et al., 2014; Yu et al., 2016). Additionally, stroke in combination with EE leads to an increased vascularization in the striatum (Ohab et al., 2006). In this context, it has been shown that angiogenesis is coupled with neurogenesis in brain tissue repair and remodeling after ischemic stroke (Kojima et al., 2010). The vasculature is critical in the migration of neuroblasts into the ischemic area (Ohab et al., 2006). In addition to increasing neurogenesis, exposure to an EE caused improved sensorimotor behavior (Komitova et al., 2006). However, other studies found that the combination of EE and additional running wheel training caused an increase in post-stroke thalamic atrophy and a deterioration of motor performance (Risedal et al., 1999). However, the timing and intensity of motor training can also worsen the functional outcome, which has already been described in previous studies (Kozlowski et al., 1996). The use of skilled reach training alone was sufficient to improve grasping after sensorimotor lesions. In 18-month-old animals, wheel training showed an improvement in motor function after endothelin-1-induced stroke. Neurogenesis was not changed (Maldonado et al., 2008).

In addition to exposure to an EE in the acute phase after the stroke, which leads to a neuroprotective effect and improve functional outcome, Tang et al. (2019) also showed a positive effect if the animals were transferred to the EE with a delay. In addition, the group showed an increased migration of cells from the SVZ toward the ischemic striatum. The study can thus indicate that exposure to an EE outside the critical phase also contributes to the recovery of the sensorimotor system. The use of the hind- and forelimbs, such as through wheel training, induced a decrease in the neuronal progenitor pool in the SVZ (Dahlqvist et al., 2003). In contrast, forced use of the lesioned forepaw (constraint-induced movement therapy, CIMT) after stroke induced increased production of neuroblasts in the SVZ and survival of newborn neurons. It is possible that the increased production of neuroblasts by forced limb use is due to reduced apoptosis. CIMT was shown to improve motor function after stroke in old animals in addition to increasing neuroblast numbers (Qu et al., 2015). Interestingly, (Liang et al., 2019) demonstrated that increased activation in the peri-infarct area by CIMT (forepaw use) induces post-stroke migration of neuroblasts and increases neurogenesis. However, if hind limbs are used then migration of progenitors is reduced. Here, the involvement of the different motor cortical areas appears to be decisive for the direction of migration.

Using the Nestin-δ-HSV-TK-EGFP transgenic mouse model, Sun et al. (2013) were able to reduce the of number progenitor cells in the SVZ and in the SGZ. This ablation did not affect motor function after stroke, although this response may also be related to the relatively low ablation rate obtained through this method, which reduced the occurrence of type 1 NPCs in SVZ by 32.1% while in the SGZ by 58.3%. The extent to which neurogenesis is related to post-ischemic functional recovery during rehabilitative training remains unclear. Studies showed a close correlation between an increase of neuroblasts in the area of the SVZ and an improved sensorimotor function especially when animals were kept in an EE or/and received specific skilled reaching training. Running alone or in combination tended to show no further improvement.

Considering the abundance of different interventions, start and end points of rehabilitative training, a diffuse picture of the cellular changes emerges. Due to the variety of neuronal and glial changes after rehabilitative training, a direct correlation is difficult, especially since ablation of SVZ precursors did not show any deterioration in sensorimotor function (Keiner et al., 2008; Sun et al., 2013). Moreover, in the EE, it is not clear which components contribute predominantly to the improvement of sensorimotor function. Basically, it will be an interplay of all the pieces that fit together like a puzzle.

### Rehabilitative Training and Dentate Neurogenesis

The relationship between adult neurogenesis and improved cognitive performance through additional rehabilitative training after ischemia has not yet been conclusively clarified (Table 1). While some studies describe positive effects of combining stroke and rehabilitation (Luo et al., 2007), other studies show no improvement or rather a deterioration of cognitive performance. The rehabilitative training paradigms used in studies were primarily exposure to an EE, providing a running wheel, and skilled or forced reaching training. Combinations of EE with running wheel or with additional reaching training were also used. All three paradigms differ in their activation of the affected body parts. While the EE provides a non-specific stimulating environment with an additional social component, running trains all extremities and reaching mainly trains the affected forelimb.

**TABLE 1:** Effect of physical activity on neurogenesis and cognitive function after stroke.

References	Model	Physical activity	Histological and functional outcome
Komitova et al., 2002	MCAO	4 weeks enriched environment starting 1 or 7 days after stroke induction	Increase in the number of astrocytes rather than number of neurons Cognition and sensorimotor function were not studied
Briones et al., 2005	4-vessel occlusion and reperfusion	2 weeks treadmill running starting 3 days after stroke induction	No change in neurogenesis (BrdU^+^NeuN^+^ cells) Cognition and sensorimotor function were not studied
Yagita et al., 2006	4-vessel occlusion and reperfusion	3 weeks voluntary running starting 7 days after stroke induction	Decrease in BrdU^+^ cells Cognition and sensorimotor function were not studied
Luo et al., 2007	MCAO	4 weeks forced swimming or voluntary running starting 7 days after stroke induction	Forced swimming: decrease in cell proliferation and improved spatial memory Voluntary running: increased BrdU^+^ cells and improved spatial memory
Wurm et al., 2007	Photothrombotic stroke	Skilled reaching training or enriched environment staring 1 day after stroke induction	Skilled reaching training: increased neurogenesis (BrdU^+^NeuN^+^ cells) and improved spatial memory and motor function Enriched environment: increased neurogenesis (BrdU^+^NeuN^+^ cells) and improved spatial memory and motor function
Geibig et al., 2012	Photothrombotic stroke	5 weeks enriched environment or voluntary running starting directly after stroke induction	Increased neurogenesis (BrdU^+^NeuN^+^ cells) Improved spatial learning
Woitke et al., 2017	MCAO	7 weeks voluntary running starting directly after stroke induction	Aberrant neurogenesis (GFP-retrovirus) Impaired spatial memory No benefit of increased neurogenesis (EdU^+^NeuN^+^ cells)
Cuartero et al., 2019	MCAO	3 weeks voluntary running starting 7 days after stroke induction	Increased Ki 67^+^ and DCX^+^ cells Aberrant neurogenesis (GFP-retrovirus) Impaired spatial/contextual memory Memory improved upon pharmacological/genetical reduction of post-stroke neurogenesis (DCX^+^)
Sun et al., 2019	MCAO	4 weeks treadmill training starting 3 days after stroke induction	Enhanced neural stem/progenitor cell proliferation, differentiation, and migration Improved sensorimotor and cognitive function
Zhai and Feng, 2019	MCAO	3 weeks constraint-induced movement therapy starting 7 days after stroke induction	Increased neurogenesis (BrdU^+^NeuN^+^ cells) Improved spatial learning
Codd et al., 2020	Unilateral hippocampal injury using vasoconstrictor endothelin-1	21 days voluntary running starting 8 days after stroke induction	Increased DCX^+^ cells Improved spatial learning
Hong et al., 2020	Photothrombotic stroke	28 days treadmill training starting 1 day after stroke induction	Increased neurogenesis (BrdU^+^DCX^+^ cells) Improved both short memory and motor function

Numerous studies have shown that keeping animals in an EE increases neurogenesis (Kempermann and Gage, 1999), and cognitive behavior (Fischer, 2016) under physiological conditions. After stroke, the EE caused increased dendritic growth, enhanced sensorimotor recovery, increased synaptic plasticity and production of trophic factors, as well as reduced apoptosis (Chen et al., 2017). Post-ischemic transfer of rodents to the EE also significantly increased adult neurogenesis compared to animals in standard cages (Geibig et al., 2012). Thus, the EE is a non-invasive external stimulus which can trigger many internal changes. Many studies have shown that EE can affect proliferation of progenitor’s cells, cell survival, in addition to improvement of cognitive behavior (Fischer, 2016).

Komitova et al. (2002) examined whether timing of EE exposure after stroke influences the outcome: rats were exposed to EE either early (24 h) or late (7 days) after a stroke. The EE was equipped with numerous exploratory objects but did not contain a running wheel. abGCs cells were counted and phenotyped after 4 weeks. It was found that early and late exposure to an EE did not affect surviving cells or neurogenesis in the ipsilateral granular cell layer compared to stroke without any interventions. However, it increased newly formed astrocytes, and this increase was even more significant in the latter group (Komitova et al., 2002). Despite increased astrogenesis, delayed exposure to EE improves functional outcomes. In one study, a modified neurological severity score (mNSS) was used to assess functional deficits, and spatial cognitive performance was tested using the Morris water maze. An improvement in spatial memory was observed 27–36 days after MCAO, accompanied by an improvement in mNSS over time after MCAO. In the same study, an increase in neuronal precursors was demonstrated by EE, indicating an increase in adult neurogenesis (Tang et al., 2019). Further studies by Grégoire et al. (2014) reinforced the finding that running and EE activate distinct, non-overlapping transcriptional networks. Wurm et al. (2007) demonstrated that both daily reaching training and transfer in an EE of rats increased neurogenesis in the dentate gyrus after focal cerebral infarctions. The extent of neurogenesis did not differ significantly between the two activity groups. The number of new neurons correlated with performance in the Morris water maze. Matsumori et al. (2006) investigated the influence of an EE and daily sensorimotor activity in a running wheel on endogenous progenitor cells in the dentate gyrus in rats after occlusion of the middle cerebral artery (MCAO). After an observation period of 8 weeks, there was a significantly increased cell survival and neuronal differentiation by physical activity (Matsumori et al., 2006). Another study by Briones et al. (2005) dealt with the influence of physical activity on neurogenesis in the dentate gyrus after global ischemia. In this study, physical activity after cerebral ischemia did not increase neurogenesis. Briones et al. (2005) suggest that physical activity after an ischemic insult does not affect progenitor cell proliferation but predominantly survival and, in part, neuronal differentiation.

The effect of rehabilitation through physical exercise is susceptible to different factors such as type, duration, and intensity of the exercise, in addition to the time delay between stroke induction and the beginning of the rehabilitation process. Although physical exercises increase NSCs proliferation, cognitive behavior as well as cell survival in intact healthy brains (Dong et al., 2019), the full effects of wheel running and stroke on neurogenesis and cognitive function are not yet completely understood. The additional increase in post-stroke neurogenesis following running is well documented (Sun et al., 2019) and long-lasting (Luo et al., 2007) up to the third week after ischemia, but some studies found conflicting results. Beneficial effects of exercise on hippocampal neurogenesis were not observed in an ischemia model in spontaneous hypertensive rats (SHR) after transient forebrain ischemia induced through 4-vessel occlusion. The number of BrdU-labeled cells in the SGZ and GCL 1 day and 14 days after BrdU administration were counted to assess neurogenesis based on co-localization of BrdU and DCX, NeuN and β-III-tubulin. Running for a period of 14 days was found to increase BrdU labeled cells in sham, but to decrease them in ischemic rats (Yagita et al., 2006). Long-term running (24 days) was also found to decrease cell proliferation in the SGZ. The functional consequences of the putative increase in running-induced neurogenesis post-stroke are also not clear. Geibig et al. (2012) showed that wheel running increases the rate of neurogenesis after stroke compared to animals with stroke under standard conditions. Interestingly, this increase in adult neurogenesis correlated with a deterioration in learning performance in the Morris Water Maze. On the other hand, Briones et al. (2005) found that 5 min of treadmill running for 2 weeks did not decrease neurogenesis, but 30 min for 3 weeks increased neurogenesis and improved short term memory. Wheel running training increases not only the number of newly generated neurons, but also the number of aberrant neurons generated in the post-stroke environment. Interestingly, despite a further boost of running-induced neurons to the already increased neurogenesis following stroke, no significant cognitive improvement in the Morris Water Maze could be found (Woitke et al., 2017), indicating failure to compensate for the cognitive deficits.

A recent study comprehensively investigated both the extent of aberrant neurogenesis post-stroke neurogenesis as well as its correlation to functional performance of mice in contextual and spatial memory paradigms (Cuartero et al., 2019). Using an MCAO model of stroke, Cuartero et al. (2019) found that mice show persistent, and possibly progressive, hippocampus-dependent memory deficits up to 60 days after the ischemic event. Interestingly, both the volume of the GCL, as well as the number of post-stroke DCX^+^ GCs correlated negatively with cognitive outcome, suggesting a deleterious effect on memory function through the abrupt addition of post-ischemic abGC. Both the ipsilateral and the contralateral hemispheres responded similarly, with an increase in adult neurogenesis peaking at about 2 weeks and returning to baseline by 65 days after stroke. The authors used running and memantine to further increase SGZ neurogenesis after stroke, which aggravated cognitive deficits. Morphological examinations revealed alterations in neuronal morphology in a subset of about 30% of post-stroke abGC, such as an ipsilateral decrease and a contralateral increase in dendritic length. Further consolidating the link between aberrant neurogenesis and post-stroke cognitive disfunction, the authors reduced aberrant neurogenesis pharmacologically (temozolomide) and genetically, which improved memory performance in mice after MCAO. The work of Cuartero et al. (2019) presents a persuasive argument for a maladaptive, deleterious effect of the enhanced post-stroke neurogenesis and, together with previous research, supports a therapeutic role for therapies suppressing neurogenesis in the treatment of post-stroke cognitive dysfunction. Taking a translational perspective, studies in subacute stroke survivors using high-intensity aerobic exercise protocols to improve cognitive performance also did not find encouraging results (Tang et al., 2016; Pallesen et al., 2019).

In summary, modulation of post-stroke adult neurogenesis through environmental (e.g., EE, running or both), pharmacological or genetical approaches can be readily achieved, but conflicting results were found with respect to its functional outcome. Methodological aspects may play a significant role and highlight the complexities of study design. While several studies found a positive effect of post-stroke neurogenesis on cognitive outcome, recent results challenge that conclusion, and suggest a maladaptive role.

## Conclusion

Numerous studies in animals have shown promising results in using enhanced neurogenesis to improve functional outcome after stroke, but the translational perspective is currently not so encouraging. The very presence of adult neurogenesis in the human brain is hotly debated, while at the same time many fundamental questions about the functional implications of enhanced post-stroke neurogenesis remain. Such questions include to what extent neuroblasts in the human brain can migrate from the neurogenic niche of the SVZ into the striatum or distant cortex and to what extent DCX^+^ neuroblasts contribute to functional recovery after stroke. Stroke-dependent neurological recovery in humans is considered to have a non-linear, logarithmic dynamic (Kwakkel et al., 2006). In this context, much of the recovery was shown to occur within the first 3 months after stroke (Wade et al., 1983). Within this time window, numerous plasticity processes of post-stroke regeneration and compensation take place involving relearning and adaptation strategies (Kwakkel et al., 2004), and adult neurogenesis may also play an important role. The difficulties of investigating adult neurogenesis in humans aside, preclinical experiments pose several technical difficulties which may impede further progress. The plethora of possible experimental variables results in very heterogenous outcomes. Dissecting the interdependent effects of stroke, niche-specific effects on adult neurogenesis and nice-specific effects on outcomes, as well as the regional-dependent effects of functional outcomes (sensorimotor or cognitive) creates a prohibitive level of complexity in study design. Of paramount importance is using the appropriate tools for the measurement of functional outcome, such as tests specifically used to assess hippocampal dependent memory (Figure 3). Stroke models, as well as interventions to modulate neurogenesis after stroke (non-invasive such as running or EE, pharmaceutical or genetical) can have effects varying in both intensity and spatial distribution. Furthermore, the developmental stages during neurogenesis (such as proliferation, differentiation, survival, maturation, or integration) where pathophysiological conditions, intervention strategies and pharmacological manipulations take place should optimally be considered, investigated and described in future studies.

Taking all the evidence into account, a niche-specific effect of neurogenesis after stroke on functional outcomes seems likely, with striatal neurogenesis having a positive effect while hippocampal neurogenesis is still not clearly understood as it can have both a positive and negative effect on recovery. Despite the recent advances in our understanding of the functional abnormalities in post-stroke abGC, many questions still remain, such as the time dynamics of these changes, how they affect the network, and the molecular mechanisms that cause them. Therapeutic strategies could also look to refine the suppression of adult neurogenesis to aberrant neurons (morphologically or functional).

## Author Contributions

MC, MD, and SK conceptualized and wrote the manuscript. OW conceptualized and edited the manuscript. All authors contributed to the article and approved the submitted version.

## Conflict of Interest

The authors declare that the research was conducted in the absence of any commercial or financial relationships that could be construed as a potential conflict of interest.

## Publisher’s Note

All claims expressed in this article are solely those of the authors and do not necessarily represent those of their affiliated organizations, or those of the publisher, the editors and the reviewers. Any product that may be evaluated in this article, or claim that may be made by its manufacturer, is not guaranteed or endorsed by the publisher.
